# IgE Sensitization Profiles Differ between Adult Patients with Severe and Moderate Atopic Dermatitis

**DOI:** 10.1371/journal.pone.0156077

**Published:** 2016-05-26

**Authors:** Irene Mittermann, Gustav Wikberg, Catharina Johansson, Christian Lupinek, Lena Lundeberg, Reto Crameri, Rudolf Valenta, Annika Scheynius

**Affiliations:** 1 Christian Doppler Laboratory for the Development of Allergen Chips, Medical University of Vienna, Vienna, Austria; 2 Division of Immunopathology, Department of Pathophysiology and Allergy Research, Center for Pathophysiology, Infectiology and Immunology, Medical University of Vienna, Vienna, Austria; 3 Department of Medicine Solna, Karolinska Institutet, and Dermatology and Venereology Unit, Karolinska University Hospital, Stockholm, Sweden; 4 Department of Clinical Science and Education, Karolinska Institutet, and Sachs' Children and Youth Hospital, SE-118 83 Södersjukhuset, Stockholm, Sweden; 5 Swiss Institute of Allergy and Asthma Research (SIAF), University of Zurich, Davos, Switzerland; CNRS-University of Toulouse, FRANCE

## Abstract

**Background:**

Atopic dermatitis (AD) is a complex chronic inflammatory disease where allergens can act as specific triggering factors.

**Aim:**

To characterize the specificities of IgE-reactivity in patients with AD to a broad panel of exogenous allergens including microbial and human antigens.

**Methodology:**

Adult patients with AD were grouped according to the SCORAD index, into severe (n = 53) and moderate AD (n = 126). As controls 43 patients were included with seborrhoeic eczema and 97 individuals without history of allergy or skin diseases. Specific IgE reactivity was assessed in plasma using Phadiatop^®^, ImmunoCap^™^, micro-arrayed allergens, dot-blotted recombinant *Malassezia sympodialis* allergens, and immune-blotted microbial and human proteins.

**Results:**

IgE reactivity was detected in 92% of patients with severe and 83% of patients with moderate AD. Sensitization to cat allergens occurred most frequently, followed by sensitization to birch pollen, grass pollen, and to the skin commensal yeast *M*. *sympodialis*. Patients with severe AD showed a significantly higher frequency of IgE reactivity to allergens like cat (rFel d 1) and house dust mite (rDer p 4 and 10), to *Staphylococcus aureus*, *M*. *sympodialis*, and to human antigens. In contrast, there were no significant differences in the frequencies of IgE reactivity to the grass pollen allergens rPhl p 1, 2, 5b, and 6 between the two AD groups. Furthermore the IgE reactivity profile of patients with severe AD was more spread towards several different allergen molecules as compared to patients with moderate AD.

**Conclusion:**

We have revealed a hitherto unknown difference regarding the molecular sensitization profile in patients with severe and moderate AD. Molecular profiling towards allergen components may provide a basis for future investigations aiming to explore the environmental, genetic and epigenetic factors which could be responsible for the different appearance and severity of disease phenotypes in AD.

## Introduction

Atopic dermatitis (AD) is a complex chronic inflammatory skin disease with 15–30% of children and 2–10% of adults being afflicted [1]. The pathogenesis of AD is considered to result from a combination of a defective skin barrier and inappropriate immune responses with contribution of both genetic and environmental factors including microbial agents [2–4]. Allergens are important in the pathogenesis of AD since they can act as specific trigger factors. For example, exacerbations of AD can be observed in patients during allergen exposure[5] or upon ingestion of allergen-containing food [6]. AD has according to the presence or to the absence of detectable allergen-specific IgE antibodies been classified into either an extrinsic or an intrinsic type [7]. Still, another subgroup of patients with AD has an autoimmune IgE-mediated reactivity against auto-antigens in addition to sensitization against exogenous allergens [8,9].

In addition microorganisms play a role in eliciting and maintaining eczema in patients with AD [10]. One such microorganism is *Malassezia*, a commensal yeast that dominates the human fungal skin flora[11] but is also associated with a number of skin disorders, such as seborrhoeic eczema (SE), dandruff and AD [12]. *Malassezia sympodialis* is one of the most frequently isolated species from the skin of both AD patients and healthy individuals [13,14]. Allergen specific IgE- and T-cell reactivity and/or positive atopy patch test reaction to *M*. *sympodialis* can be detected in around 50% of adult patients with AD[12]. These reactions are rarely found in other allergic diseases or skin disorders indicating a specific link between AD and *Malassezia* in a subset of patients [15]. So far there are 10 *M*. *sympodialis* allergens, designated Mala s 1 and Mala s 5–13, cloned, sequenced and characterized [16–22]. Four of the allergens lack homology to known proteins, whereas others have cross-reactivity to human homologues such as manganese superoxide dismutase [19] and thioredoxin [21]. The uniqueness of these allergens makes them highly interesting as diagnostic tools and to understand sensitization and cross-reactivity to human homologous antigens in AD.

There are several techniques for determining IgE sensitizations such as allergen-specific IgE serology, skin prick test, and when necessary oral food challenges. By introducing new methodologies for phenotype characterization of patients with AD it may be possible to develop personalized strategies for prevention and management to break, terminate or reverse the natural course of this chronic disease. One way of achieving this goal could be using micro-arrayed allergen molecules which allow measuring IgE reactivities in only a few microliters of serum or plasma to large panels of purified allergen molecules to identify disease-causing allergens [23,24].

In this study we sought to perform a meticulous analysis of the IgE-reactivity profiles in AD patients with different severity to obtain information if and how molecular IgE sensitization profiles towards a comprehensive set of exogenous, microbial and endogenous allergens may be linked with different clinical phenotypes of AD including co-morbidity with respiratory symptoms.

## Materials and Methods

### Study population

All subjects (n = 319) were recruited from the Stockholm area and examined by a dermatologist at the Dermatology and Venereology Unit, Karolinska University Hospital Stockholm, Sweden, during September until May to avoid the summer season. Patients with a clinical diagnosis of AD or SE and healthy controls were included in the study (Table 1). Inclusion criteria for AD patients were diagnosis according to the UK working party [25], moderate to severe eczema, and skin lesions not only restricted to the hands. The severity of the eczema was assessed using the objective SCORAD index [26] and present or patient reported head and neck distribution was recorded. Exclusion criteria for the patients were other skin diseases than AD or SE, immune deficiencies, autoimmune diseases and ongoing malignant diseases. The history of patients reported respiratory allergic symptoms was documented as co-morbidity. The healthy controls were subjects who did not have clinical symptoms or history of allergy or skin disease. The study was approved by the Regional Ethical Review Board in Stockholm and all participants gave their written informed consent.

**Table 1:** Demographic and clinical characterization of AD patients and controls.

		Gender F / M	Age (years)	SCORAD ^a)^	Head and neck distribution	Rhino-conjunctivitis and/or asthma	Total plasma IgE ^b)^ ≥122 kU/L	Total plasma IgE ^b)^ (kU/L)	Phadiatop^®^ positive ^c)^
	n	n	median (range)	median (range)	n (%)	N (%)	N (%)	median (range)	n (%)
AD patients	179	101/ 78	28 (18–65)	34 (14–70)	156 (87)	144 (80)	102 (57)	175 (2–15100)	140 (78)
Severe ^a)^	53	19 / 34	30 (18–65)	54 (41–70)	48 (91)	48 (91)	38 (72)	480 (2.5–15100)	46 (87)
Moderate ^a)^	126	82/ 44	28 (18–65)	31 (14–40)	108 (85)	96 (76)	64 (51)	130 (2–6810)	94 (75)
SE patients	43	9 / 34	39 (18–63)	NA	NA	9 (21)	4 (9)	22 (2–390)	8 (19)
Healthy controls	97	59 / 38	35 (18–65)	NA	NA	0 (0)	5 (5)	21 (2–230)	9 (9)

^a)^ Objective SCORAD [26], severe AD defined as SCORAD ≥41

^b)^ ImmunoCAP^™^ (Phadia AB), reference range 1.6–122 kU/L

^c)^ Phadiatop^®^ (Phadia AB), plasma IgE to any of 11 common aeroallergens, reference range ≥ 0.35 kU/L

AD = atopic eczema, F = Female, M = Male, n = number of positive individuals, NA = not applicable, SE = seborrhoeic eczema

### Preparation of protein/allergen extracts

*M*. *sympodialis* (ATCC strain 42132) and human A431 epithelial cell extracts were prepared as previously described [8,27]. *Staphylococcus aureus* (ATCC 25923) was grown overnight in tryptic soy broth at 37°C and harvested by centrifugation at 3220 x g. The cells were washed twice with phosphate-buffered saline (PBS) and inactivated by boiling for 10 min. The cell pellet was re-suspended in PBS and homogenized using an Ultra Turrax (IKA Labortechnik, Staufen, Germany) and stored at -20°C until use.

### Recombinant allergens

Recombinant Mala s 1, rMala s 5–13 (S1 Table) were expressed in *Escherichia coli* BL21 (DE3) as his-tagged proteins and purified by inclusion body preparation and Ni^2+-^chelate chromatography [16–21]. Purity of proteins was assessed by SDS-PAGE and Coomassie Blue staining [28]. Protein concentrations were determined by Micro BCA Protein Assay (Pierce, Rockford, IL).

### IgE serology

Total IgE, specific IgE to any of 11 common aeroallergen sources (Phadiatop^®^) and *M*. *sympodialis*-specific IgE (m70,) were measured in plasma using ImmunoCAP^™^ (Phadia AB, Uppsala, Sweden).

For non-denaturing RAST-based dot blot assays, 0.2 μg purified recombinant allergens were dotted onto nitrocellulose membrane strips (Schleicher&Schuell, Dassel, Germany) and tested for IgE-reactivity with ^125^I-labelled anti-human IgE antibodies (Demeditec Diagnostics, Kiel, Germany) [29]. For IgE immunoblot assays, comparable amounts (50 μg protein extract per centimeter slot of the preparative gel) of *M*. *sympodialis*, human A431 epithelial cell line or *S*. *aureus* extracts were boiled for 5 min with sodium dodecyl sulphate (SDS) sample buffer containing 5% v/v beta-mercaptoethanol, subjected to 12.5% SDS-PAGE and blotted onto nitrocellulose membranes [30,31]. A protein molecular weight marker (PageRuler prestained Protein Ladder Plus, Fermentas, St Leon-Rot, Germany) was used as standard. To define a cut-off for a positive/negative dot blot and immunoblot result human serum albumin (HSA) and serum from non-allergic patients were used for control purposes. In every experiment three sera from AD patients containing specific IgE to *S*. *aureus*, *M*. *sympodialis* and the human extract were included as positive control.

IgE-reactivity to 120 micro-arrayed allergen components was analyzed with a customized allergen-chip (Phadia Multiplexing, Thermo Fisher Scientific, Vienna, Austria) as previously described [24].

### Statistical analyses

Descriptive statistics and group comparisons were computed using IBM SPSS Statistics, version 22 (IBM Corp., Armonk, NY, USA). For comparison of the two groups of patients suffering from moderate or severe AD, respectively, with respect to frequencies of reactivities to bacterial, yeast and human antigens and to micro-arrayed allergen molecules, two-sided asymptotic p-values were calculated by Chi-square test. P-values ≤ 0.05 were considered statistically significant.

## Results

### Demographic and clinical characterization of AD patients and controls

The 179 AD patients (56% females) had a median age of 28 years (range 18–65 years) (Table 1). According to SCORAD 53 patients (30%) suffered from severe AD and 156 patients (87%) had present (n = 143) or previous (n = 13) head and neck distribution of their eczema. Debut of the eczema was similar in both groups; in the majority of the AD patients the debut was during the first year (58% in the severe group and 57% in the moderate group) and 17% and 22%, respectively, after 5 years of age. The majority of AD patients (i.e., 144, 80%), had ever had rhino-conjunctivitis and/or asthma in addition to AD (i.e., 91% in the severe and 76% in the moderate AD group) whereas only 21% of the SE patients reported respiratory symptoms (Table 1).

Total plasma IgE levels were elevated (>122 kU/L) in 57% of the patients, with higher median IgE levels in the severe AD group (480 kU/L) than in the moderate AD group (130 kU/L) (Table 1). Seventy-eight % of the AD patients had a positive Phadiatop^®^ with a higher percentage among the severe AD patients (87%) in comparison to the moderate AD patients (75%) (Table 1). Nine % of 43 individuals with SE had a total serum IgE level >122 kU/L and 19% had a positive Phadiatop^®^ (Table 1). Five % of the healthy controls (n = 97) had IgE levels higher than 122 kU/L and for 9% the Phadiatop^®^ was positive (Table 1).

### IgE reactivity to bacterial (*S*. *aureus)*, yeas*t (M*. *sympodialis*) and human antigens was significantly more frequent in the severe AD group

IgE reactivity to nitrocellulose-blotted *S*. *aureus*, *M*. *sympodialis* and human antigens (epithelial cell-line A431) was analyzed using plasma samples from all 179 AD patients, 43 SE patients and 97 healthy controls. Twenty-one percent of the AD patients showed reactivity to antigens in the *S*. *aureus* extract (i.e., 34% in the severe AD group compared to 16% in the moderate AD group, p<0.01 (Table 2). Eighteen percent of the AD patients showed IgE reactivity to the human cell extract. Again the percentage of reactivity was significantly higher (i.e., 30%) in severe than in moderate AD patients (i.e., 13%) (p<0.01) (Table 2).

**Table 2:** Frequencies of allergen-specific IgE reactivities in the AD and SE patients as tested by MeDALL allergen chip, Immunoblotting, ImmunoCAP^™^ and RAST-based dot-blot assay.

	All AD	Severe AD	Moderate AD	SE patients
Method and allergen	n = 179	n = 53	n = 126	n = 43
	n (%)	n (%)	n (%)	n (%)
**MeDALL allergen chip** positive^a)^ n (%)	146 (82)	45 (85)	101 (80)	10 (23)
**Immunoblot extracts**				
*S*. *aureus* positive^b)^ n (%)	38 (21)	18 (34) **^g)^	20 (16)	0 (0)
A431 positive^c)^ n (%)	32 (18)	16 (30) **	16 (13)	0 (0)
*M*. *sympodialis* positive^d)^ n (%)	81 (45)	32 (60) **	49 (39)	1 (2)
**ImmunoCAP**^**™**^				
*M*. *sympodialis* positive^e)^ n (%)	85 (47)	33 (62) **	52 (41)	0 (0)
Median (range) kU/L within positive individuals	6.7 (0.44–230)	7.5 (0.44–68)	6.7 (0.47–230)	-
**Dot-Blot, rMala s allergens** positive n (%)				
rMala s 1	18 (10)	6 (11)	12 (10)	0 (0)
rMala s 5	27 (15)	9 (17)	18 (14)	0 (0)
rMala s 6	28 (16)	11 (21)	17 (13)	0 (0)
rMala s 7	25 (14)	13 (25) **	12 (10)	0 (0)
rMala s 8	4 (2)	2 (4)	2 (2)	0 (0)
rMala s 9	11 (6)	6 (11)	5 (4)	0 (0)
rMala s 10	21 (12)	9 (17)	12 (10)	0 (0)
rMala s 11	28 (16)	12 (23)	16 (13)	1 (2)
rMala s 12	14 (8)	6 (11)	8 (6)	0 (0)
rMala s 13	54 (30)	20 (38)	34 (27)	0 (0)
Any rMala s^f)^	78 (44)	31 (59)	47 (37)	1 (2)

^a)^ MeDALL allergen chip, positive ≥ 0.30 ISU

^b)^ *Staphylococcus aureus* ATCC 25923 extract

^c)^ A431 Human epithelial cell line extract

^d)^ *Malassezia sympodialis* ATCC 42132 extract

^e)^ ImmunoCAP^™^ (Phadia AB), *Malassezia sympodialis* ATCC 42132 extract (m70), positive ≥0.35 kU/L

^f)^ Individuals positive to one or more of the tested rMala s allergens

^g)^ Significant differences between severe and moderate AD patients are indicated ** p<0.01

AD = atopic dermatitis, SE = seborrhoeic eczema

Likewise, IgE reactivity against the *M*. *sympodialis* extract was detected significantly more often in severe (i.e., 60%) compared to moderate AD patients (i.e., 39%) with 45% of the 179 AD patients being positive on average (p<0.01) (Table 2). In addition, specific IgE to *M*. *sympodialis* was assessed with the ImmunoCap^™^ assay where 47% of the 179 AD patients, 62% with severe and 41% with moderate eczema (p<0.01) (Table 2 and S1A–S1C Fig), whereas none of the SE patients (Table 2) or healthy controls was positive in this assay (S2 Table). The median specific plasma IgE levels against *M*. *sympodialis* of the severe AD patients was 7.5 kU/L and of the moderate AD patients 6.7 kU/L (Table 2). The majority, 98% (n = 156) of AD patients with IgE reactivity to *M*. *sympodialis* had present or reported head and neck involvement (S2 Table). Notably, 8% in the group of 53 severe AD patients had a negative Phadiatop^®^ reaction, not elevated total serum IgE and no detectable IgE antibodies to *M*. *sympodialis*, m70 (S1B Fig), and among the 126 patients with moderate AD the corresponding figure was 19% (S1C Fig). There was a good agreement of results obtained with the immunoblot and ImmunoCAP^™^ technology, only slightly higher positive results were observed with the CAP assay compared to the immunoblotting method (AD 47% compared to 45%, severe AD 62% versus 60%, moderate AD 41% versus 39%, Table 2).

No IgE-binding to *S*. *aureus* or human A431 extract was observed among the 43 SE patients, but one of them who was Phadiatop^®^ positive, showed a weak IgE reactivity to one band in the *M*. *sympodialis* extract (Table 2). None of the 97 healthy controls was positive in these assays.

We then performed an analysis of IgE reactivity to a set of 10 purified, recombinant Mala s allergens (Mala s 1, Mala s 5–13, S1 Table) using a non-denaturing, RAST-based dot blot assay (Table 2). IgE-reactivity to the different rMala s allergens varied between 2–30% among the AD patients, with higher percentages for each of the allergens in the severe AD group (Table 2) and with 4–67% IgE-reactivity among the AD patients ImmunoCAP^™^ or immunoblot positive to the *M*. *sympodialis* extract (S2 Table). When reactivity to any of the 10 rMala s allergens was summarized 44% of all AD patients were detected yielding an almost equal sensitivity as the allergen extract-based ImmunoCAP^™^ (47%) or Immunoblot assay (45%) (Table 2). IgE-reactivity to rMala s 7 was significantly more frequently detected in severe compared to moderate AD patients (p<0.01, Table 2). rMala s 13 showed IgE reactivity in 38% of the severe and 27% of the moderate AD patients, respectively, and was the most frequently detected Mala s allergen (Table 2). Among the 85 *M*. *sympodialis* ImmunoCAP^™^ positive AD patients 52 of them (61%) reacted to rMala s 13 (S2 Table), therefore to be considered as a major allergen.

Only 1 patient with SE showed IgE reactivity to rMala s 11 (Table 2) and one healthy control to rMala s 13 (S2 Table).

### A limited number of allergen sources contribute to a different extent to sensitization in severe and moderate AD patients

Using the allergen chip [24] with 120 allergen components (S3 Table), 82% of the 179 AD patients showed IgE reactivity to at least one allergen, 85% of the severe and 80% of the moderate AD patients were positive, respectively, and 23% of the 43 patients with SE showed IgE-reactivity to micro-arrayed allergens (Table 2).

**Fig 1:**
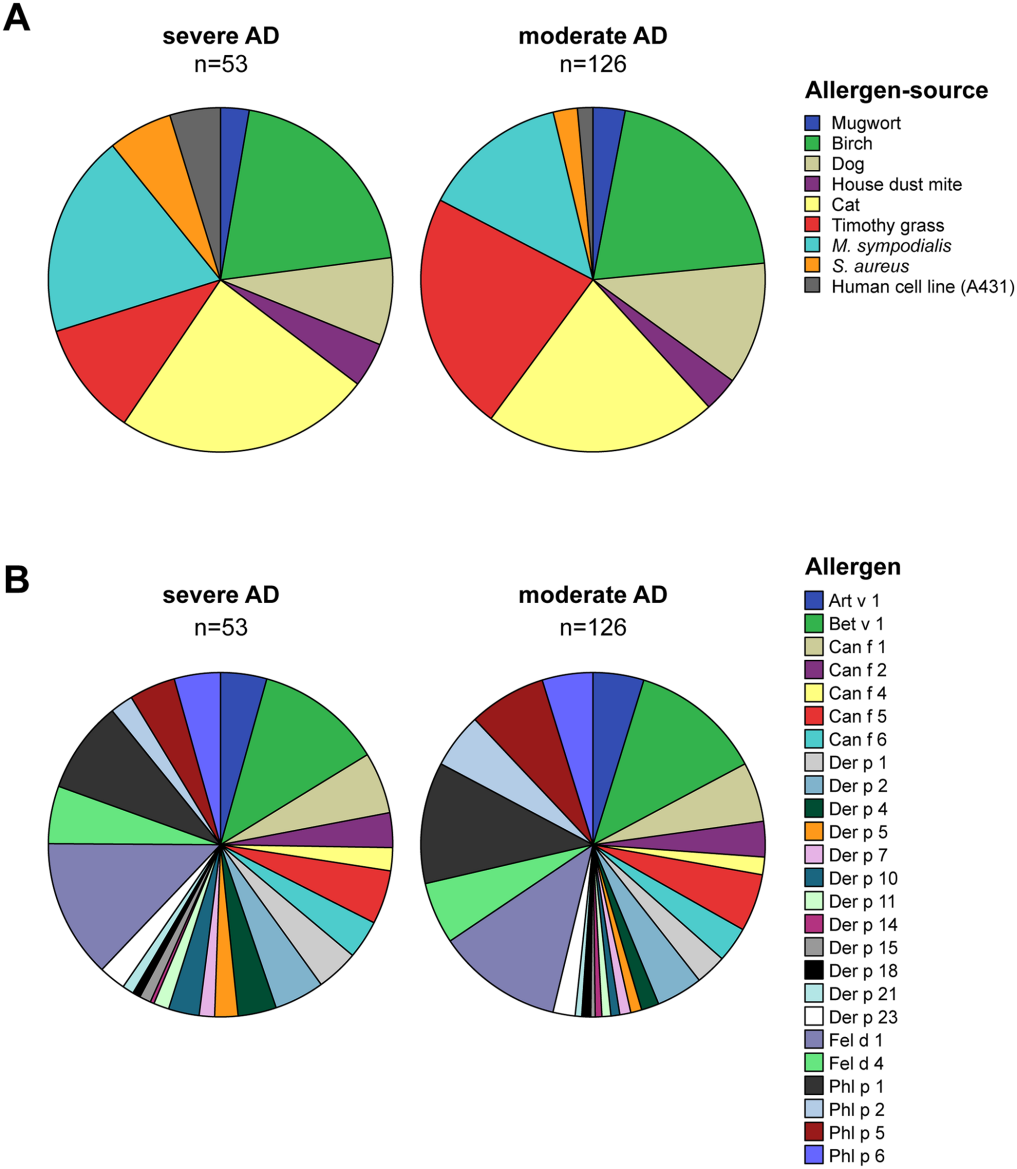
Pie charts showing the contribution of (A), allergen sources and (B), individual allergen components to IgE sensitization. Each chart represents 100% of IgE reactivities detected in plasma from all AD patients of the respective group, severe AD (left chart) and moderate AD (right chart), in (**A)** to six allergen sources using the MeDALL allergen-chip (ISU ≥ 0.3) and to extracts of *M*. *sympodialis*, *S*. *aureus* and the human cell line A431 using immunoblotting, and in (**B)** to 25 allergen molecules using the MeDALL allergen-chip (ISU ≥ 0.3). The sizes of the segments represent the proportion of the respective in (**A)**, allergen source and in (**B)**, allergen molecule among all recognized. Allergen sources/molecules start a 12 o’clock of the pie chart and continue clock-wise as listed with the color code.

Fig 1A shows a pie chart representation of the contribution of different allergen sources to IgE sensitization in severe and moderate AD patients. The chart was constructed based on the frequencies of IgE sensitizations towards *M*. *sympodialis*, *S*. *aureus* and human cell extracts detected with immunoblotting (Table 2) and based on IgE-reactivity profiles towards 25 specific allergen components determined with micro-arrayed allergen molecules (Table 3 and S3 Table). As mentioned above the IgE sensitization to *M*. *sympodialis*, *S*. *aureus* and human antigens contributed significantly more to IgE sensitization in severe versus moderate AD (p<0.01, Table 2). IgE sensitization to genuine cat allergens (rFel d 1 and/or rFel d 4) and house dust mite allergens (nDer p 1 and/or rDer p 2, rDer p 4, rDer p 5, rDer p 7, rDer p 10, rDer p 11, rDer p 14, rDer p 15, rDer p 18, rDer p 21, rDer p 23) also contributed more to IgE sensitization in severe versus moderate AD (Fig 1A). Birch pollen (rBet v 1) and mugwort (nArt v 1) contributed equally to IgE sensitization in severe and moderate AD but interestingly, the contribution of IgE sensitization to grass pollen (rPhl p 1 and/or rPhl p 2, rPhl p 5b, rPhl p 6) and dog allergens (rCan f 1 and/or rCan f 2, rCan f 4, rCan f 5, rCan f 6) was less in severe as compared to moderate AD (Fig 1A). IgE sensitization to other allergen sources was quite rare (S3 Table). Only 10% of all AD patients showed IgE reactivity to latex allergens (rHev b 6.01) and 2% to the mould *Alternaria alternata* (rAlt a 1). IgE reactivities to recombinant food allergens, e.g., apple (rMal d 1, 42%), peach (rPru p 1, 18%), hazelnut (rCor a 1.0401, 34%) or soy (rGly m 4, 16%) were due to IgE cross-reactivity to the major birch pollen allergen Bet v 1 (52%) (S3 Table). Other plant-derived food allergens, e.g., hazelnut (rCor a 8), kiwi allergens (nAct d 1, nAct d 5) or wheat allergens (rTri a 19.0101) were recognized by only 4%, or less of the AD patients. IgE to animal-derived food allergens could be detected in only a small percentage of the AD patients, e.g., 7% of all AD patients showed IgE reactivity to egg allergens and cow`s milk allergens were recognized by 2% of the patients. The frequencies of IgE reactivity to each of the individual micro-arrayed allergens are reported in S3 Table.

**Table 3:** Frequencies and intensities of IgE reactivity to purified marker allergens in AD patients detected by allergen chip technology.

No.	Allergen	All AD	Severe AD	Moderate AD	Severe AD	Moderate AD	Allergen family, Function, CCD	Severe AD	Moderate AE
					median (range)	median (range)		Rhinoconjuctivitis	Rhinoconjuctivitis
		n = 179	n = 53	n = 126	IgE (ISU)^a)^	IgE (ISU)		and/or asthma	and/or asthma
		n (%)	n (%)	n (%)				n = 47 n (%)	n = 96 n (%)
1	rFel d 1	93 (52)	36 (68) **	57 (45)	4.3 (0.31–134.17)	4.14 (0.35–127.92)	Uteroglobin	36 (77) **	51 (53)
2	rBet v 1	93 (52)	33 (62)	60 (48)	12.83 (0.33–120.37)	8.96 (0.34–102.94)	PR-10	32 (68)	51 (53)
3	rPhl p 1	79 (44)	24 (45)	55 (44)	2.11 (0.31–48.74)	2.43 (0.31–56.91)	β-Expansin	23 (49)	45 (47)
4	nPhl p 4	51 (29)	22 (42) *	29 (23)	1.53 (0.31–16.6)	1.25 (0.39–26.31)	Grass-group 4, CCD	21 (45) *	26 (27)
5	rPhl p 5b	47 (26)	12 (23)	35 (28)	4.83 (0.61–28.58)	3.41 (0.32–86.9)	Grass-group 5	11 (23)	32 (33)
6	rCan f 1	43 (24)	16 (30)	27 (21)	14.28 (0.44–111.13)	6.91 (0.61–121.54)	Lipocalin	16 (34)	25 (26)
7	rFel d 4	43 (24)	15 (28)	28 (22)	9.32 (0.45–118.56)	2.9 (0.34–55.56)	Lipocalin	15 (32)	27 (28)
8	rCan f 5	40 (22)	14 (26)	26 (21)	2.29 (0.54–28.17)	3.62 (0.35–27.48)	Arginine Esterase	14 (30)	23 (24)
9	rPhl p 6	35 (20)	12 (23)	23 (18)	0.89 (0.38–14.56)	2.66 (0.36–33.03)	Grass-group 5/6	11 (23)	21 (22)
10	nArt v 1	35 (20)	12 (23)	23 (18)	1.14 (0.34–43.7)	1.39 (0.32–38.99)	Defensin-like protein	12 (26)	21 (22)
11	rDer p 2	34 (19)	13 (25)	21 (17)	33.79 (0.57–131.74)	5.18 (0.35–103.86)	Group 2 mite-allergen	12 (26)	20 (21)
12	rPhl p 2	31 (17)	6 (11)	25 (20)	9.72 (0.74–27.15)	5.59 (0.36–33.3)	Grass-group 2/3	5 (11) *	24 (25)
13	rCan f 6	26 (15)	10 (19)	16 (13)	2.11 (0.4–14.01)	1.72 (0.3–7.25)	Lipocalin	10 (21)	15 (16)
14	nDer p 1	25 (14)	11 (21)	14 (11)	4.99 (0.62–18.63)	0.88 (0.4–31.58)	Group 1 mite- allergen	10 (21)	13 (14)
15	rCan f 2	25 (14)	9 (17)	16 (13)	1.03 (0.4–49.04)	2.09 (0.34–41)	Lipocalin	9 (19)	15 (16)
16	rDer p 4	18 (10)	10 (19) *	8 (6)	0.87 (0.32–3.57)	0.71 (0.32–4.87)	Group 4 mite-allergen	9 (19) *	7 (7)
17	rDer p 23	18 (10)	7 (13)	11 (9)	29.98 (0.7–98.11)	14.16 (1.69–43.88)	Chitin-binding domain	6 (13)	11 (12)
18	rCan f 4	14 (8)	6 (11)	8 (6)	8.65 (0.45–64.14)	12.25 (0.39–25.9)	Lipocalin	6 (13)	7 (7)
19	rDer p 10	12 (7)	8 (15) **	4 (3)	0.49 (0.3–3.8)	2.57 (0.44–3.47)	Tropomyosin	7 (15) *	3 (3)
20	rDer p 5	11 (6)	6 (11)	5 (4)	1.48 (0.44–93.01)	2.47 (0.34–87.36)	Group 5 mite-allergen	6 (13)	4 (4)
21	rDer p 7	9 (5)	4 (8)	5 (4)	1.82 (0.37–3.71)	11.65 (0.75–22.5)	Group 7 mite-allergen	4 (9)	4 (4)
22	rDer p 11	8 (4)	4 (8)	4 (3)	0.42 (0.3–1.62)	0.86 (0.31–1.09)	Group 11 mite-allergen	3 (6)	4 (4)
23	rDer p 15	5 (3)	3 (6)	2 (2)	0.33 (0.32–16.44)	0.7 (0.68–0.71)	Chitin-binding domain	3 (6)	2 (2)
24	rDer p 21	6 (3)	3 (6)	3 (2)	2.52 (0.86–71.48)	47.28 (11.23–80.02)	Group 21 mite-allergen	3 (6)	3 (3)
25	rDer p 18	6 (3)	2 (4)	4 (3)	3.76 (1.85–5.67)	1.21 (0.32–8.22)	Chitin-binding domain	2 (4)	4 (4)
26	rDer p 14	4 (2)	1 (2)	3 (2)	0.68 (0.68)	0.56 (0.39–1.28)	Vitellogenin	1 (2)	3 (3)
								36 (77)	

^a)^ MeDALL allergen chip, reference range ≥ 0.3 ISU

Significant differences between severe and moderate AD patients are indicated * p<0.05, ** p<0.01. Taken into account the number of tested variables (n = 26) the expected number of allergens to be significant at p level <0.05 by chance is 1.3.

AD = atopic dermatitis, CCD = cross-reactive carbohydrate determinates, n = natural, r = recombinant

In the group of 43 SE-patients, 14% showed IgE-reactivity to birch pollen (rBet v 1), 14% to grass pollen (rPhl p 1), 5% to house dust mite (rDer p 2) and 2% to dog allergens (rCan f 1) (S3 Table). Plasma samples from 12 Phadiatop^®^ negative healthy controls were tested for control purposes and they were all negative (S3 Table).

### Selective spreading in the molecular sensitization profiles of severe as compared to moderate AD patients

The frequencies of IgE reactivity to the individual allergen molecules are shown in Table 3 for the most frequently recognized allergen sources and for each of the tested components in S3 Table. For most of the allergens we observed that they were recognized more frequently by patients with severe as compared to patients with moderate AD (Table 3, S2A, S2B and S2D–S2H Fig). For certain allergens (e.g. rFel d 1, rDer p 4, and rDer p 10) a significantly (p<0.05) more frequent IgE recognition was found for the severe AD patients (Table 3). Interestingly, genuine grass pollen allergens (i.e., rPhl p 1, rPhl p 2, rPhl p 5b, and rPhl 6) did not follow this trend because they were recognized with a similar frequency in both patient groups (Table 3, S2C Fig). Only the cross-reactive carbohydrate marker allergen from grass pollen (i.e., nPhl p 4) was more frequently recognized by severe AD patients (p<0.05, Table 3, S2C Fig). The same pattern was observed when the patients were grouped into severe and moderate AD with patient reported history of respiratory allergic symptoms, with the exception that a significant difference (p<0.05) was reached for a higher IgE reactivity to rPhl p 2 in the moderate AD patients compared to the severe AD patients (Table 3).

The levels of allergen-specific IgE to the individual allergens expressed in ISU varied between severe and moderate AD patients. For some allergens (e.g., rFel d 1) the IgE levels were comparable and for others severe AD patients showed higher allergen-specific IgE levels (e.g., rFel d 4, rDer p 2, rDer p 23) (Table 3).

An interesting finding was that the IgE reactivity profile of patients with severe AD was more spread towards several different allergen molecules as compared to patients with moderate AD (Fig 1B). The latter becomes visible when the contribution of the individual allergens to IgE sensitization is displayed in the form of a pie chart where it becomes obvious that the segment of the 12 house dust mite allergens occupy a larger part, 26%, of the pie in severe AD patients compared to 17% in moderate AD patients (Fig 1B). In contrast the contribution of grass pollen allergens (i.e., rPhl p 1, rPhl p 2, rPhl p 5b and rPhl p 6) is lower in severe AD patients 19% compared to 29% in the moderate AD group (Fig 1B).

### Summary of allergen-specific IgE reactivities in the AD patients

Altogether, IgE reactivity as tested to 120 allergen components (MeDALL allergen chip), and to *M*. *sympodialis*, *S*. *aureus* and human epithelial cell extracts (immunoblotting assays) was detected in 92% of patients with severe and 83% of patients with moderate AD (Table 4). Six % (n = 3) of the severe and 12% (n = 15) of the moderate AD patients were mono-sensitized. The three patients in the severe group were all mono-sensitized to *M*. *sympodialis* whereas the sensitization profile in the moderate group was spread over seven different allergen sources. The majority of the patients were sensitized to 2–25 allergens/allergen components (Table 4). The maximum number of allergens/allergen components detected in a patient in the severe group was 55 and in the moderate group 48.

**Table 4:** Summary of allergen-specific IgE reactivities in the AD patients.

	All AD	Severe AD	Moderate AD
IgE reactivity to the allergens/allergen components tested ^a)^	n = 179	n = 53	n = 126
	n (n%)	n (n%)	n (n%)
Any allergen/allergen component	154 (86%)	49 (92%)	105 (83%)
Mono-sensitized ^b)^	18 (10%)	3 (6%)	15 (12%)
Sensitized to 2–25 allergens/allergen components	109 (61%)	34 (64%)	75 (59%)
Sensitized to 26–55 allergens/allergen components	27 (15%)	12 (22%)	15 (12%)

^a)^ Tested for IgE reactivity to 120 allergen components (MeDALL allergen chip), and to *M*. *sympodialis*, *S*. *aureus* and human epithelial cell extracts (immunoblotting).

^b)^ Mono-sensitised severe AD patients: *M*. *sympodialis* (n = 3)

Mono-sensitised moderate AD patients: Grass pollen (n = 2), birch pollen (1), dog (1), cat (5), house dust mite (1), *M*. *sympodialis* (4), wasp venom (1).

## Discussion

In this study we used a comprehensive panel of allergen molecules from exogenous allergen sources, microbes and autoantigens to characterize the molecular IgE reactivity profile of AD patients with defined clinical phenotypes. We found that patients with severe differed from patients with moderate AD regarding several characteristics. First, severe AD patients were characterized by the fact that they reacted to a larger panel of environmental allergens than patients with moderate AD. Interestingly, the spreading of the IgE recognition profiles in the severe AD patients included allergens from certain environmental allergen sources such as house dust mite, cat, and birch pollen allergens but not the major timothy grass pollen allergens (Phl p 1, Phl p 5, Phl p 2 and Phl p 6) (Fig 1). This finding would suggest that grass pollen allergens are less important as trigger factors for AD compared to birch pollen and indoor allergens in the studied population. The difference between birch pollen and grass pollen is unexpected because grass pollen contains several very potent allergens whereas birch contains only one major allergen (Bet v 1). Furthermore, the grass pollen season lasts longer than the birch pollen season and allergen loads are typically higher for grass pollen [32,33]. Since the route of exposure should be the same for both pollens it is quite conceivable that other factors play a role such as climatic effects where low humidity and cold temperatures negatively affect skin barrier functions and increase the risk of dermatitis [34]. We therefore hypothesize that an impaired skin barrier of patients after the usually long winter periods in Sweden before the birch pollen season may be responsible for the fact that birch pollen is associated more with severe AD whereas the skin barrier may regenerate towards the later grass pollen season.

Secondly, patients suffering from severe AD also differed from patients with moderate AD regarding their recognition of microbial “allergens” and autoantigens. IgE-reactivity to skin-associated microorganisms like *M*. *sympodialis* or *S*. *aureus* as well as to human antigens was significantly higher in the severe AD group (Table 2). The percentage in all AD patients who showed IgE-reactivity to the *M*. *sympodialis* extract, as detectable with ImmunoCap^™^ or immunoblot analyses, was 47–45%, which is in agreement with previous studies as is the high frequency of head and neck involvement, particularly in the severe AD patients (S2 Table) [15,35–37].

Regarding IgE reactivity to the 10 rMala s allergens using Dot blot analyses, the dominating allergen was rMala s 13 (30%) which has sequence similarities to human thioredoxin [21]. Evidence for T cell–mediated cross-reactivity between human thioredoxin, and Mala s 13 has been demonstrated, suggesting that thioredoxin-autoreactive skin-homing T cells might contribute to the pathogenesis of AD by perpetuating skin inflammation and chronification of eczema in patients with AD sensitized to *Malassezia* [38]. The AD patients, who in addition to reactivity to environmental allergens like *M*. *sympodialis*, also have cross-reactivity to human counterparts, are highly interesting from a pathogenic aspect. Besides Mala s 13, Mala s 6 (a cyclophilin), and Mala s 11 (a manganese-dependent superoxide dismutase) share a high degree of sequence identity to the corresponding human enzymes (S1 Table) and might therefore play an essential role in perpetuating skin inflammation of AD due to cross-reactivity. It is thus quite possible that IgE recognition of human homologous allergens may have been triggered by sensitization to cross-reactive microbial allergens.

Different explanations for the broader and more spread allergen profile recognized by the severe AD patients may be considered. On the one hand it is possible that the severity of AD may depend on the number of different allergen molecules recognized and thus the sum of recognized allergens may play a role as has been recently reported for respiratory allergy to house dust mites: Children with more severe respiratory manifestations (i.e., asthma plus rhinitis) exhibited a broader IgE-recognition towards individual house dust mite allergens than children suffering only from rhinitis [39]. This is similar to the findings made: We also found a broader recognition of house dust mite allergens in the severe AD group as compared to the moderate AD group (Table 3 and Fig 1). In this context, it has also been discovered that AD patients differ regarding their molecular recognition profiles of house dust mite allergens compared to patients suffering only from house dust mite-associated respiratory allergy [40].

Alternatively, one may consider also other explanations. For example it may be considered that patients with more severe AD and broader IgE recognition profiles may be more “atopic” than the patients with moderate AD. It has been reported that patients with poly-sensitization to respiratory allergens differed from patients with oligo/monosensitization by their production of IL-4 and Th2 driving cytokines [41]. In this scenario which was recently suggested as a hypothesis [42], a more “atopic” genetic make-up would be responsible for poly-sensitization and for a more severe disease. This is in agreement with our study where besides having higher IgE levels, more broadly spread sensitization pattern and higher frequency of IgE-reactivity, 91% of the AD patients in the severe group reported co-morbidity with respiratory allergic symptoms compared with 76% in the moderate AD group (Table 1). Finally, one must consider epigenetic modifications as additional factors for the development of different disease phenotypes.

Another highly interesting subgroup of AD patients are those with a clinical picture of AD but who lack detectable IgE antibodies to known allergens and have normal total serum IgE levels. In the present study including all allergen components tested 13% of the AD patients were found in this category (8% of patients with severe and 16% of patients with moderate AD). At present, one may therefore define this group as “intrinsic” but it is possible that the corresponding allergens triggering the skin inflammation in this group are not yet defined. Alternatively, it is quite possible that these patients suffer from non-IgE-associated forms of AD [43].

In summary, our study revealed a hitherto unknown difference regarding the molecular sensitization profile in patients with severe and moderate AD. Molecular profiling towards allergen components may thus provide a basis for future investigations aiming to explore the environmental, genetic and epigenetic factors which could be responsible for the different appearance and severity of disease phenotypes in AD leading to a platform for future prevention and treatment strategies of different AD subgroups.

## Supporting Information

S1 Fig Concordance between total serum IgE, positive Phadiatop^®^ and specific serum IgE to *M*. *sympodialis* extract using ImmunoCAP^™^. (**A**) 179 AD patients, (**B**) 53 severe AD patients, and (**C**) 126 moderate AD patients.
(TIF)

S2 Fig **Frequencies and intensities of IgE reactivity to**: (**A)**, members of the PR-10 allergen family, (**B)**, animal allergens, (**C)**, grass pollen allergens, (**D)**, house dust mite allergens, (**E)**, plant pollen allergens, (**F)**, cow’s milk and egg allergens, (**G)**, nut allergens, and (**H)**, latex and mould allergens in patients with severe (s), moderate (m) atopic dermatitis (AD) or seborrhoeic eczema (SE) measured by allergen chip technology. Displayed are percentages (*y-axes*) of sera containing IgE in the range of 0.3 to 0.99 ISU (white boxes), 1 to 15 ISU (grey) and >15 ISU (black) to the respective allergens (*x-axes*).
(TIF)

S1 Table Recombinant *M*. *sympodialis* allergens used in this study. (PDF)

S2 Table *M*. *sympodialis* specific IgE reactivity in AD patients and controls. (PDF)

S3 Table Frequencies of IgE reactivity to purified allergens in AD patients and controls detected by allergen chip technology. (PDF)

## Abbreviations

AD: Atopic dermatitis
ISU: ISAC standardized units
PBS: Phosphate-buffered saline
SCORAD: Scoring atopic dermatitis
SDS: sodium dodecyl sulphate
SE: Seborrhoeic eczema
