# Supplementary figures and images for: IgE Sensitization Profiles Differ between Adult Patients with Severe and Moderate Atopic Dermatitis
Source: PLoS One. 2016 May 26;11(5):e0156077. doi: 10.1371/journal.pone.0156077 (PMC4881900; doi:10.1371/journal.pone.0156077)

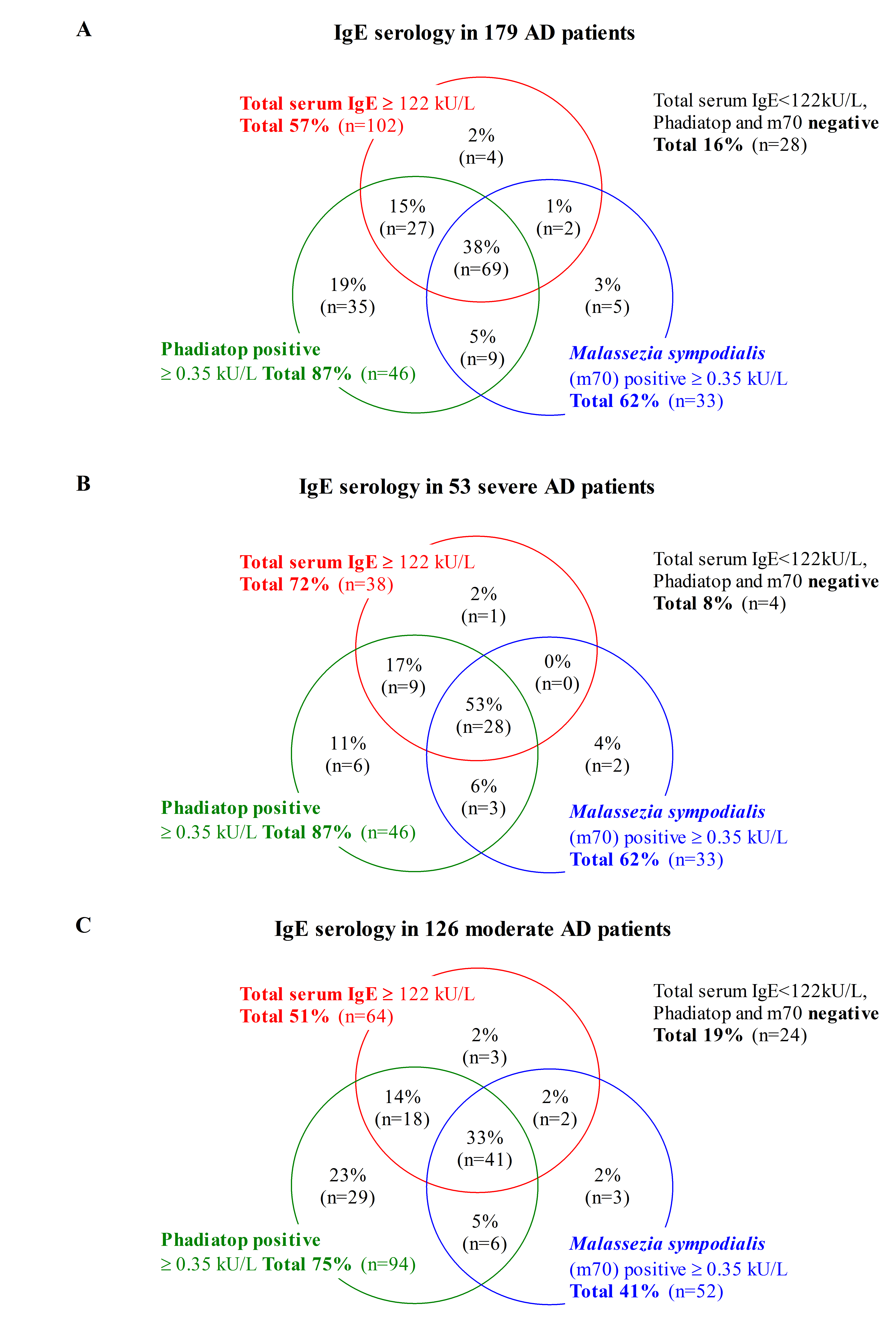

Supplement: S1 Fig — (A) 179 AD patients, (B) 53 severe AD patients, and (C) 126 moderate AD patients. (TIF) [file pone.0156077.s001.tif]

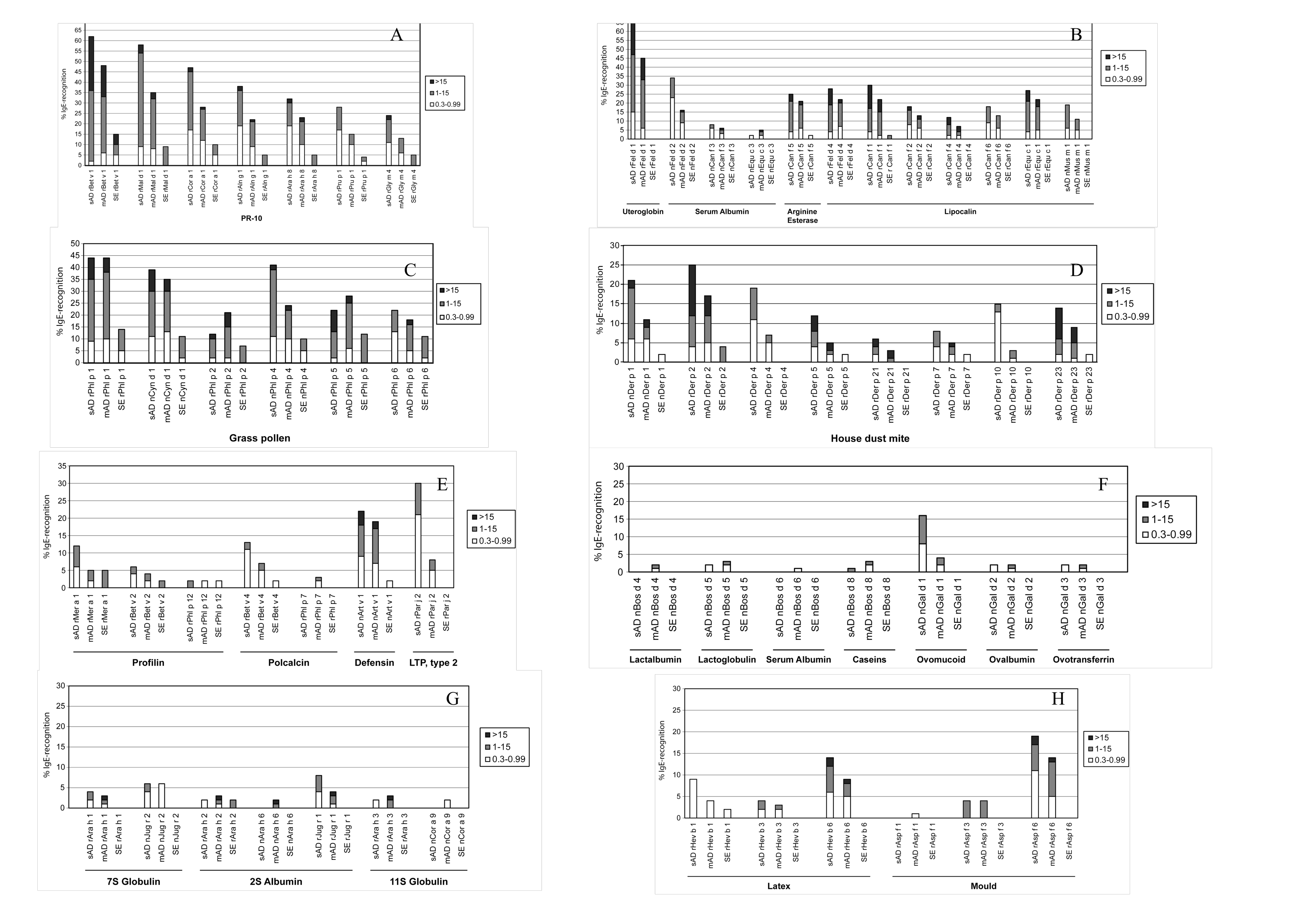

Supplement: S2 Fig — Frequencies and intensities of IgE reactivity to: (A), members of the PR-10 allergen family, (B), animal allergens, (C), grass pollen allergens, (D), house dust mite allergens, (E), plant pollen allergens, (F), cow’s milk and egg allergens, (G), nut allergens, and (H), latex and mould allergens in patients with severe (s), moderate (m) atopic dermatitis (AD) or seborrhoeic eczema (SE) measured by allergen chip technology. Displayed are percentages (y-axes) of sera containing IgE in the range of 0.3 to 0.99 ISU (white boxes), 1 to 15 ISU (grey) and >15 ISU (black) to the respective allergens (x-axes). (TIF) [file pone.0156077.s002.tif]
