# Supplementary material for: IgE Sensitization Profiles Differ between Adult Patients with Severe and Moderate Atopic Dermatitis
Source: PLoS One. 2016 May 26;11(5):e0156077. doi: 10.1371/journal.pone.0156077 (PMC4881900; doi:10.1371/journal.pone.0156077)
Supplement: S1 Table — (PDF) [file pone.0156077.s003.pdf]

S1 Table. Recombinant *M. sympodialis* allergens used in this study

**Allergen**	**Accession number** [reference]	**Function**	**Prediction for Secretion** [20]	**Cross-reactivity to human protein**
Mala s 1	X96486 [16]	unknown	yes	no
Mala s 5	AJ011955 [17]	peroxisomal protein	no	No
Mala s 6	AJ011956 [17]	cyclophilin	no	Yes
Mala s 7	AJ011957 [18]	unknown	yes	No
Mala s 8	AJ011958 [18]	unknown	yes	No
Mala s 9	AJ011959 [18]	unknown	no	No
Mala s 10	AJ428052 [19]	heat shock protein	no	Yes
Mala s 11	AJ548421 [19]	manganese superoxide dismutase	no	Yes
Mala s 12	AJ871960 [20]	glucose-methanol-choline oxidoreductase	yes	Yes
Mala s 13	AJ937746 [21]	thioredoxin	no	Yes
