# Supplementary material for: IgE Sensitization Profiles Differ between Adult Patients with Severe and Moderate Atopic Dermatitis
Source: PLoS One. 2016 May 26;11(5):e0156077. doi: 10.1371/journal.pone.0156077 (PMC4881900; doi:10.1371/journal.pone.0156077)
Supplement: S2 Table — (PDF) [file pone.0156077.s004.pdf]

S2 Table. *Malassezia* specific IgE reactivity in AD patients and controls

Method and allergen	All AD patients	AD patients positive to *M. sympodialis* ImmunoCAP	AD patients positive to *M. sympodialis* Immunoblot	Severe AD (SCORAD ≥41) [a]		Moderate AD (SCORAD ≤40) [a]		SE patients	Healthy controls
				Head and neck involvement	Without head and neck involvement	Head and neck involvement	Without head and neck involvement		
	N = 179	N = 85	N = 81	N = 48	N = 5	N = 108	N = 18	N = 43	N = 97
	n (%)	n (%)	n (%)	n (%)	n (%)	n (%)	n (%)	n (%)	n (%)
**ImmunoCAP™**									
M. sympodialis [b]	85 (47)	85 (100)	74 (91)	32 (67)	1 (20)	51 (47)	1 (6)	0 (0)	0 (0)
**Immunoblot**									
M. sympodialis [c]	81 (45)	74 (87)	81 (100)	31 (65)	1 (20)	48 (44)	1 (6)	1 (2)	0 (0)
**Dot Blot**									
rMala s 1 [d]	18 (10)	18 (21)	18 (22)	6 (13)	0 (0)	12 (11)	0 (0)	0 (0)	0 (0)
rMala s 5 [d]	27 (15)	26 (31)	25 (31)	9 (19)	0 (0)	18 (17)	0 (0)	0 (0)	0 (0)
rMala s 6 [d]	28 (16)	28 (33)	28 (35)	11 (23)	0 (0)	17 (16)	0 (0)	0 (0)	0 (0)
rMala s 7 [d]	25 (14)	24 (28)	24 (30)	13 (27)	0 (0)	12 (11)	0 (0)	0 (0)	0 (0)
rMala s 8 [d]	4 (2)	3 (4)	3 (4)	2 (4)	0 (0)	2 (2)	0 (0)	0 (0)	0 (0)
rMala s 9 [d]	11 (6)	11 (13)	11 (14)	6 (13)	0 (0)	5 (5)	0 (0)	0 (0)	0 (0)
rMala s 10 [d]	21 (12)	20 (24)	20 (25)	9 (19)	0 (0)	11 (10)	1 (6)	0 (0)	0 (0)
rMala s 11 [d]	28 (16)	27 (32)	27 (33)	12 (25)	0 (0)	15 (14)	1 (6)	1 (2)	0 (0)
rMala s 12 [d]	14 (8)	14 (16)	14 (17)	6 (13)	0 (0)	8 (7)	0 (0)	0 (0)	0 (0)
rMala s 13 [d]	54 (30)	52 (61)	54 (67)	19 (40)	1 (20)	34 (31)	0 (0)	0 (0)	1 (1)
Any rMala s [e]	79 (44)	73 (86)	74 (91)	31 (65)	1 (20)	48 (44)	1(6)	1 (2)	1 (1)

a) Objective SCORAD [26], severe AD defined as SCORAD ≥41
b) ImmunoCAP™ (Phadia AB), *M. sympodialis* ATCC 42132 extract (m70), positive ≥0.35 kU/L
c) *M. sympodialis* ATCC 42132 extract
d) Recombinant *M. sympodialis* allergens
e) Individuals positive in one or more of the tested rMala s allergens

AD=atopic eczema, N=number of individuals, n=number of positive individuals, SE=seborrhoeic eczema
