# Supplementary material for: IgE Sensitization Profiles Differ between Adult Patients with Severe and Moderate Atopic Dermatitis
Source: PLoS One. 2016 May 26;11(5):e0156077. doi: 10.1371/journal.pone.0156077 (PMC4881900; doi:10.1371/journal.pone.0156077)
Supplement: S3 Table — (PDF) [file pone.0156077.s005.pdf]

S3 Table. **Frequencies of IgE reactivity to purified allergens in AD patients and controls detected by allergen chip technology**

No.	Allergen	All AD n=179	Severe AD n=53	Mode-rate AD n=126	SE patients n=43	Healthy controls n=12	Allergen family, Function, CCD
1	rFel d 1	52%	68%	45%	0%	0%	Fel d 1 family, Uteroglobin
2	rBet v 1	52%	62%	48%	14%	0%	PR-10
3	rPhl p 1	44%	45%	44%	14%	0%	Grass group 1, β-Expansin
4	rMal d 1	42%	58%	35%	9%	0%	PR-10
5	nCyn d 1	36%	40%	34%	12%	0%	Grass group 1, β-Expansin
6	rCor a 1.0401	34%	47%	28%	9%	0%	PR-10
7	nPhl p 4	29%	42%	23%	9%	0%	Grass group 4, Berberine bridge enzyme, CCD
8	rAln g 1	26%	38%	21%	5%	0%	PR-10
9	rPhl p 5b	26%	23%	28%	12%	0%	Grass group 5
10	rAra h 8	25%	32%	22%	5%	0%	PR-10
11	rCan f 1	24%	30%	21%	2%	0%	Lipocalin
12	rFel d 4	24%	28%	22%	0%	0%	Lipocalin
13	rEqu c 1	23%	26%	22%	0%	0%	Lipocalin
14	rCan f 5	22%	26%	21%	2%	0%	Arginine Esterase
15	nFel d 2	21%	34%	16%	0%	0%	Serum Albumin
16	rPhl p 6	20%	23%	18%	12%	0%	Grass group 5/6
17	nArt v 1	20%	23%	18%	2%	0%	Defensin-like protein
18	rDer p 2	19%	25%	17%	5%	0%	Group 2 mite allergen, NPC2 family
19	rPru p 1	18%	28%	14%	5%	0%	PR-10
20	rDer f 2	17%	23%	15%	2%	0%	Group 2 mite allergen, NPC2 family
21	rPhl p 2	17%	11%	20%	7%	0%	Grass group 2/3
22	rGly m 4	16%	25%	13%	5%	0%	PR-10
23	rAsp f 6	16%	19%	14%	0%	0%	Mn superoxide Dismutase

24	rPar j 2	15%	30%	8%	0%	0%	nsLTP, type 2
25	rCan f 6	15%	19%	13%	0%	0%	Lipocalin
26	nDer p 1	14%	21%	11%	2%	0%	Group 1 mite allergen, Cysteine protease
27	rCan f 2	14%	17%	13%	0%	0%	Lipocalin
28	nDer f 1	13%	19%	11%	2%	0%	Group 1 mite allergen, Cysteine protease
29	nMus m 1	13%	19%	11%	0%	0%	Lipocalin
30	rHev b 8	11%	21%	7%	7%	0%	Profilin
31	rDer p 4	10%	19%	6%	2%	0%	Group 4 mite allergen, α-Amylase
32	rDer p 23	10%	13%	9%	2%	0%	Chitin-binding domain
33	rHev b 6.01	10%	13%	9%	0%	0%	Hevein
34	rOle e 9	9%	15%	7%	0%	0%	Glucanase
35	rBet v 4	9%	13%	7%	2%	0%	Polcalcin
36	nCup a 1	8%	13%	6%	2%	0%	Pectate Lyase, CCD
37	rPru p 3	8%	11%	6%	0%	0%	nsLTP, type 1
38	rCan f 4	8%	11%	6%	0%	0%	Lipocalin
39	rPhl p 11	8%	9%	7%	0%	0%	Ole e 1-related protein
40	rDer p 10	7%	15%	3%	0%	0%	Group 10 mite allergen, Tropomyosin
41	nGal d 1	7%	15%	3%	0%	0%	Ovomucoid
42	rMer a 1	7%	11%	5%	5%	0%	Profilin
43	nPen m 2	7%	9%	6%	0%	0%	Arginine kinase-like protein
44	rPru du 4	6%	13%	3%	5%	0%	Profilin
45	rDer p 5	6%	11%	4%	2%	0%	Group 5 mite allergen
46	rPol d 5	6%	11%	3%	4%	0%	Insect venom, Antigen 5
47	rHev b 1	6%	9%	4%	2%	0%	Rubber elongation factor
48	nCan f 3	6%	8%	6%	0%	0%	Serum Albumin
49	nJug r 2	6%	6%	6%	0%	0%	Storage protein, 7S globulin, CCD
50	rVes v 5	6%	6%	6%	2%	0%	Insect venom, Antigen 5
51	nPla a 2	5%	8%	4%	0%	0%	Polygalacturonase, CCD

52	rDer p 7	5%	8%	4%	2%	0%	Group 7 mite allergen
53	rJug r 1	5%	8%	4%	0%	0%	Storage protein, 2S albumin
54	nArt v 3	5%	8%	4%	0%	0%	nsLTP, type 1
55	rDer p 11	4%	8%	3%	0%	0%	Group 11 mite allergen, Paramyosin
56	rTri a 19.0101	4%	8%	3%	0%	0%	Storage protein, Omega 5 gliadin
57	rBet v 2	4%	6%	4%	2%	0%	Profilin
58	rCor a 8	4%	6%	4%	0%	0%	nsLTP, type 1
59	nCry j 1	4%	6%	3%	0%	0%	Pectate Lyase, CCD
60	rOle e 1	4%	4%	4%	2%	0%	Ole e 1-related protein
61	rAsp f 3	4%	4%	4%	0%	0%	Peroxysomal protein
62	nEqu c 3	4%	2%	6%	0%	0%	Serum Albumin
63	nAct d 1	3%	8%	2%	0%	0%	Cysteine protease
64	nPen m 1	3%	8%	1%	0%	0%	Tropomyosin
65	rDer p 15	3%	6%	2%	0%	0%	Chitin-binding domain
66	rDer p 21	3%	6%	2%	0%	0%	Group 21 mite allergen
67	rBla g 2	3%	6%	2%	0%	0%	Aspartic protease
68	rTri a 12	3%	4%	3%	2%	0%	Profilin
69	rDer p 18	3%	4%	3%	0%	0%	Chitin-binding domain
70	rHev b 3	3%	4%	3%	0%	0%	Small rubber particle protein
71	rAra h 1	3%	4%	2%	0%	0%	Storage protein, 7S globulin
72	rAra h 9	3%	4%	2%	0%	0%	nsLTP, type 1
73	rGad c 1	3%	4%	2%	0%	0%	Parvalbumin
74	rApi m 1	3%	4%	2%	2%	0%	Insect venom, Phospholipase A2
75	rPru du 3	2%	6%	1%	0%	0%	nsLTP, type 1
76	rAna o 3	2%	4%	1%	0%	0%	Storage protein, 2S albumin
77	rBer e 1	2%	4%	1%	0%	0%	Storage protein, 2S albumin
78	rDer p 14	2%	2%	2%	0%	0%	Vitellogenin, Apolipophorins
79	rLep d 2	2%	2%	2%	0%	0%	Group 2 mite allergen, NPC2 family

80	rBlo t 5	2%	2%	2%	0%	0%	Group 5 mite allergen
81	nGly m 5	2%	2%	2%	0%	0%	Storage protein, 11S globulin
82	nGly m 6	2%	2%	2%	0%	0%	Storage protein, 11S globulin
83	rAra h 2	2%	2%	2%	0%	0%	Storage protein, 2S albumin
84	rAra h 3	2%	2%	2%	0%	0%	Storage protein, 11S globulin
85	nBos d 5	2%	2%	2%	0%	0%	Lipocalin, β-Lactoglobulin
86	nBos d 8	2%	2%	2%	0%	0%	Caseins (α-, β-, κ-caseins)
87	rBos d 12	2%	2%	2%	0%	0%	Caseins (κ-casein)
88	nGal d 3	2%	2%	2%	0%	0%	Ovotransferrin, Conalbumin
89	rPhl p 12	2%	2%	2%	2%	0%	Profilin
90	rJug r 3	2%	0%	3%	0%	0%	nsLTP, type 1
91	rAlt a 1	2%	0%	3%	0%	0%	Acidic glycoprotein
92	rPhl p 7	2%	0%	3%	0%	0%	Calcium-binding proteins, Polcalcin
93	nBla g 7	1%	2%	1%	0%	0%	Tropomyosin
94	rAni s 3	1%	2%	1%	0%	0%	Tropomyosin
95	nOle e 7	1%	4%	0%	0%	0%	nsLTP, type 1
96	nSal k 1	1%	4%	0%	0%	0%	Pectin methylesterase
97	nSes i 1	1%	2%	1%	0%	0%	Storage protein, 2S albumin
98	rPru du 6.01	1%	2%	1%	0%	0%	Storage protein, 11S globulin
99	rAna o 2	1%	2%	1%	0%	0%	Storage protein, 11S globulin
100	nGal d 2	1%	2%	1%	0%	0%	Ovalbumin
101	rPla a 3	1%	2%	1%	0%	0%	nsLTP, type 1
102	nAct d 2	1%	2%	1%	0%	0%	Thaumatin-like protein
103	nPen m 4	1%	2%	1%	0%	0%	Calcium-binding protein
104	rTri a 14	1%	2%	0%	0%	0%	nsLTP, type 1
105	nAct d 5	1%	2%	0%	0%	0%	Kiwellin
106	rPla l 1	1%	2%	0%	0%	0%	Ole e 1-related protein
107	rBla g 1	1%	2%	0%	0%	0%	Cockroach group 1
108	rPis v 3	1%	2%	0%	0%	0%	Storage protein, 7S globulin
109	rHev b 5	1%	2%	0%	2%	0%	Acidic protein

110	nBos d 4	1%	0%	2%	0%	0%	α-Lactalbumin
111	rBos d 9	1%	0%	2%	0%	0%	Caseins (αS1-casein)
112	rBos d 10	1%	0%	2%	0%	0%	Caseins (αS2-casein)
113	rBos d 11	1%	0%	2%	0%	0%	Caseins (β-casein)
114	nAra h 6	1%	0%	2%	0%	0%	Storage protein, 2S albumin
115	nCor a 9	1%	0%	2%	0%	0%	Storage protein, 11S globulin
116	nBos d 6	1%	0%	1%	0%	0%	Serum Albumin
117	nAmb a 1	1%	0%	1%	0%	0%	Pectate Lyase
118	rAna o 1	1%	0%	1%	0%	0%	Storage protein, 7S globulin
119	rAsp f 1	1%	0%	1%	0%	0%	Mitogellin Family (Ribonuclease)
120	rBla g 5	1%	0%	1%	0%	0%	Glutathione S-transferase

a) MeDALL allergen chip, reference range ≥ 0.3 ISU
AD=atopic dermatitis, CCD=cross-reactive carbohydrate determinates, r=recombinant, n=natural, SE=seborrhoeic eczema
